# Reoperation after surgical treatment for benign prostatic hyperplasia: a systematic review

**DOI:** 10.3389/fendo.2023.1287212

**Published:** 2023-11-09

**Authors:** Weixiang He, Ting Ding, Zhiping Niu, Chunlin Hao, Chengbin Li, Zhicheng Xu, Yuming Jing, Weijun Qin

**Affiliations:** ^1^ Department of Urology, Xijing Hospital, The Fourth Military Medical University, Xi’an, China; ^2^ Department of Clinical Laboratory Medicine, Xijing Hospital, The Fourth Military Medical University, Xi’an, China; ^3^ Department of Environmental Health, School of Public Health, Fudan University, Shanghai, China

**Keywords:** benign prostate hyperplasia, lower urinary tract symptoms, surgery, retreatment, reoperation

## Abstract

**Context:**

Surgical treatment is important for male lower urinary tract symptom (LUTS) management, but there are few reviews of the risks of reoperation.

**Objective:**

To systematically evaluate the current evidence regarding the reoperation rates of surgical treatment for LUTS in accordance with current recommendations and guidelines.

**Evidence acquisition:**

Eligible studies published up to July 2023, were searched for in the PubMed^®^ (National Library of Medicine, Bethesda, MD, USA), Embase^®^ (Elsevier, Amsterdam, the Netherlands), and Web of Science™ (Clarivate™, Philadelphia, PA, USA) databases. STATA^®^ (StataCorp LP, College Station, TX, USA) software was used to conduct the meta-analysis. Random-effects models were used to calculate the pooled incidences (PIs) of reoperation and the 95% confidence intervals (CIs).

**Evidence synthesis:**

A total of 119 studies with 130,106 patients were included. The reoperation rate of transurethral resection of the prostate (TURP) at 1, 2, 3, and 5 years was 4.0%, 5.0%, 6.0%, and 7.7%, respectively. The reoperation rate of plasma kinetic loop resection of the prostate (PKRP) at 1, 2, 3, and 5 years was 3.5%, 3.6%, 5.7%, and 6.6%, respectively. The reoperation rate of holmium laser enucleation of the prostate (HoLEP) at 1, 2, 3, and 5 years was 2.4%, 3.3%, 5.4%, and 6.6%, respectively. The reoperation rate of photoselective vaporization of the prostate (PVP) at 1, 2, 3, and 5 years was 3.3%, 4.1%, 6.7%, and 7.1%, respectively. The reoperation rate of surgery with AquaBeam^®^ at 1, 2, 3, and 5 years was 2.6%, 3.1%, 3.0%, and 4.1%, respectively. The reoperation rate of prostatic artery embolization (PAE) at 1, 2, 3, and 5 years was 12.2%, 20.0%, 26.4%, and 23.8%, respectively. The reoperation rate of transurethral microwave thermotherapy (TUMT) at 1, 2, 3, and 5 years was 9.9%, 19.9%, 23.3%, and 31.2%, respectively. The reoperation rate of transurethral incision of the prostate (TUIP) at 5 years was 13.4%. The reoperation rate of open prostatectomy (OP) at 1 and 5 years was 1.3% and 4.4%, respectively. The reoperation rate of thulium laser enucleation of the prostate (ThuLEP) at 1, 2, and 5 years was 3.7%, 7.7%, and 8.4%, respectively.

**Conclusion:**

Our results summarized the reoperation rates of 10 surgical procedures over follow-up durations of 1, 2, 3, and 5 years, which could provide reference for urologists and LUTS patients.

**Systematic review registration:**

https://www.crd.york.ac.uk/PROSPERO, identifier CRD42023445780.

## Introduction

Lower urinary tract symptoms (LUTSs) related to benign prostatic hyperplasia (BPH) are very common in older men and seriously affect their quality of life (1). Although α1-adrenoceptor antagonists and 5α-reductase inhibitors are first-line drugs with good efficacy, many adverse events such as dizziness, asthenia, postural hypotension, and low libido may occur as a result of treatment with them (1). In addition, there are some patients who have poor drug responsiveness or for whom these drugs are eventually unable to delay disease progress. Therefore, many patients ultimately require surgical intervention (1). According to the current guidelines, indications of the need for surgery include renal insufficiency, refractory urinary retention, recurrent urinary tract infections (UTIs) or gross hematuria, bladder stones, or the patient being refractory to or unwilling to use other therapies (2, 3). Transurethral resection of prostate (TURP) has long been considered the “gold standard” for the surgical management of LUTSs/BPH (4). In recent decades, many new technologies and procedures have been widely used and recommended by clinical guidelines, such as plasma kinetic resection of prostate (PKRP), holmium laser enucleation of the prostate (HoLEP), and photoselective vaporization of the prostate (PVP) (2, 3). Based on the current guidelines, the most suitable type of surgery for a patient depends on their prostate volume (PV), physical condition, and economic situation, and can also even be dependent on the preference of the surgeon and the machines owned by the hospital (2, 3).

Since the physical characteristics of the surgical technique and the anatomy of the prostate vary across patients, some may suffer bladder neck contracture (BNC), urethral stricture, or other complex complications postoperatively, and these may need surgical retreatment (1). In addition, some surgical procedures do not provide patients with satisfactory relief from their symptoms, or do not prevent the reappearance of bladder outlet obstruction over time after surgery, which may also require surgical retreatment. Moreover, some recommended surgical procedures are still under investigation such as surgery with AquaBeam^®^ and prostatic artery embolization (PAE), of which the efficacy, safety, and tolerability still need to be confirmed (3). When selecting an appropriate surgical approach, knowledge of the reoperation rates could be used to predict the cost and management of disease in the years following the operation.

In the past, many studies have reported on the reoperation rate after various kinds of surgery. For patients who had undergone TURP, an Austrian nationwide study reported that the retreatment rate at the 1-year follow-up was 3.7%, and that this increased by approximately 1%–2% with each subsequent year (5, 6). A recent study reported that the rate of secondary surgery for TURP, transurethral incision of prostate (TUIP), and PVP at the 5-year follow-up was 10.3%, 13.6%, and 11.6%, respectively (7). Other procedures such as PAE and transurethral microwave thermotherapy (TUMT) were reported to have a higher risk of retreatment (8, 9). Recently, a systematic review also summarized the pharmacologic and surgical retreatment rates after newer office-based treatments, including water vapor thermal therapy (WVTT), prostatic urethral lift (PUL), and that using a temporarily implanted nitinol device (iTIND) (10). However, there is still a limited number of reviews on the reoperation rate of common surgeries recommended by the guidelines. We therefore conducted an updated systematic review and meta-analysis to summarize the reoperation rates of common surgical treatment for LUTSs/BPH. This review could be important to both BPH/LUTS patients and urologists when they are selecting an appropriate surgical procedure.

## Methods

### Literature search

This systematic review was conducted in accordance with the Preferred Reporting Items for Systematic Reviews and Meta-Analyses (PRISMA) guidelines (11). The protocol was registered in the International Prospective Register of Systematic Reviews (PROSPERO) database (registration number CRD42023445780).

Studies were searched for in the PubMed^®^ (National Library of Medicine, Bethesda, MD, USA), Embase^®^ (Elsevier, Amsterdam, the Netherlands), and Web of Science™ (Clarivate™, Philadelphia, PA, USA) databases up to July 2023. The primary outcomes were the rates of surgical retreatment during follow-up. The search strategy is provided in the **Supplementary Files**. The initial screening, which included reading the title and abstract, was performed by the two authors independently (WXH and TD). Subsequently, the full text of potentially relevant studies was acquired for further confirmation and the data extraction process. Any conflicts that arose between the two authors during article selection and data extraction were resolved through discussion with an arbitrator (ZPN).

### Inclusion and exclusion criteria

Articles that met the following criteria were included: (1) those that reported on the surgical retreatment rate of BPH/LUTS patients who had undergone operations in hospitals during the follow-up period; (2) those that were focused only on procedures recommended in the recent guidelines of the Association of University Administrators (AUA) and the European Association of Urology (EAU), including TURP, PKRP, TUIP, open prostatectomy (OP), thulium:yttrium aluminum garnet laser (Tm : YAG), enucleation of the prostate (ThuLEP), HoLEP, PVP, surgery with AquaBeam, PAE, and TUMT; (3) those that reported on a randomized controlled trial (RCT), non-randomized prospective study, or retrospective study; (4) those that were original peer-reviewed human participant research studies; (5) those that were published in English; and (6) those with a follow-up duration of 1, 2, 3, or 5 years. Studies such as reviews, editorials, commentaries, meeting abstracts of unpublished studies, and case reports were excluded. For duplicate publications, the higher-quality study, or the study that had been most recently published was selected.

### Data extraction

Data were extracted from eligible studies by the two authors independently (WXH and TD). The extracted data included the first author’s surname, publication year, country of research, study design, patient information, follow-up time, and rates of surgical retreatment. The patient information collected included the patient’s number, age, prostate volume (PV), International Prostate Symptom Score (IPSS), postvoid residual volume (PVR), and maximum urinary flow rate (*Q*_max_). It should be noted that surgical retreatment included both the management of the prostatic obstruction and of postoperative complications such as bladder neck contracture or urethral stricture. For some studies, we calculated the rate for further investigation if authors reported only the number of retreatment patients.

### Quality assessment

The risk of bias (RoB) and quality of each eligible study were assessed by two authors independently (WXH and TD). For RCTs, the RoB was assessed, summarized, and then visualized using the Cochrane Collaboration RoB tool embedded in the RevMan (The Cochrane Collaboration, The Nordic Cochrane Centre, Copenhagen, Denmark) software (version 5.4). For single-arm studies, the RoB was assessed in accordance with the EAU guidelines for systematic reviews (12).

### Data synthesis

For each surgical type, the baseline characteristics of patients were summarized and then pooled using Microsoft Excel^®^ (Microsoft Corporation, Redmond, WA, USA) software (2016). In addition, the pooled incidences (PIs) and corresponding 95% confidence intervals (CIs) of the surgical retreatment rates were evaluated and stratified by the surgical type and follow-up duration (i.e., 1 year, 2 years, 3 years, and 5 years) using STATA (version 17.0; StataCorp LP, College Station, TX, USA). A random-effects model was used to estimate the pooled incidences.

## Results

### Study selection and characteristics

**Figure 1:**
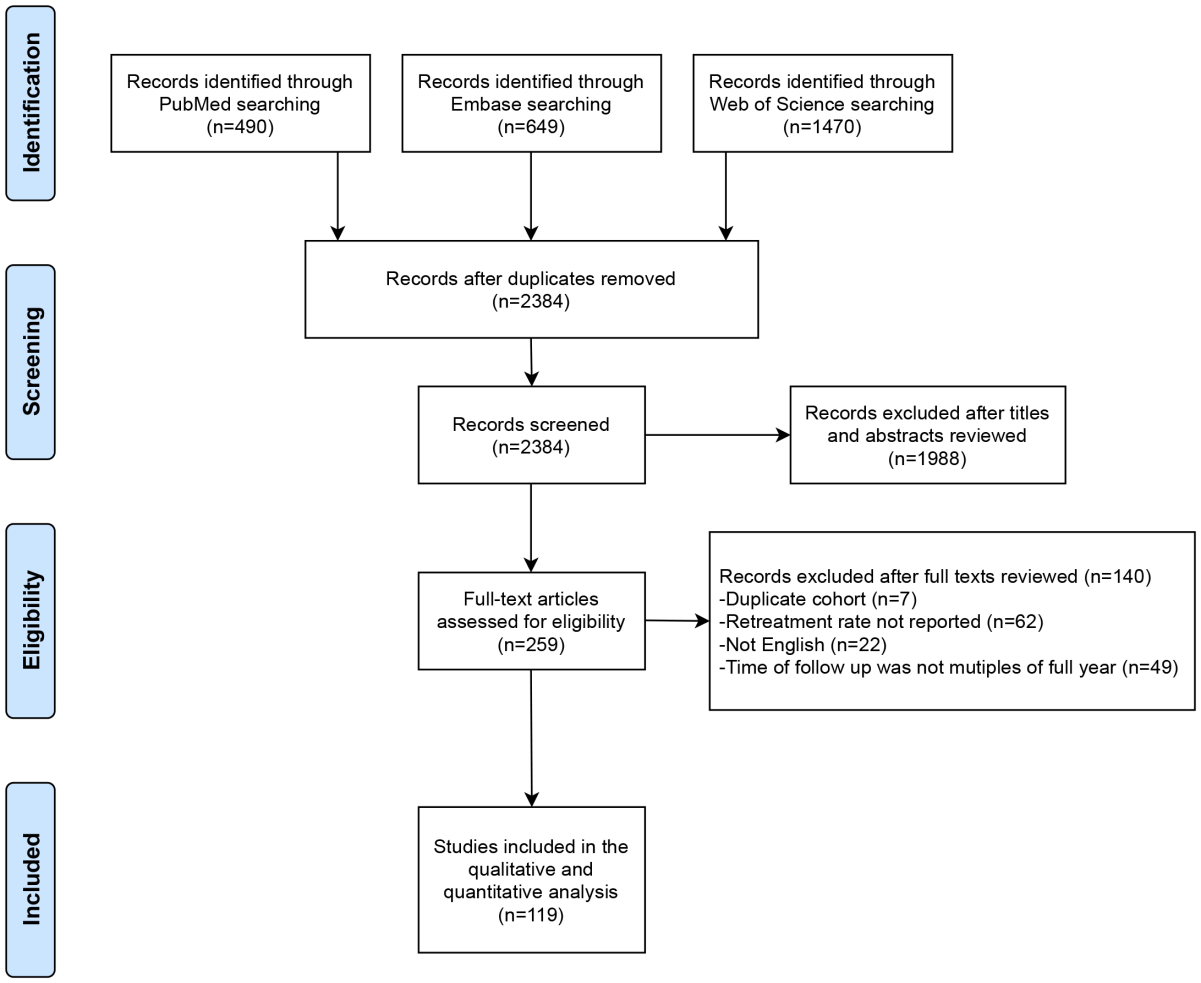
Preferred Reporting Items for Systematic Reviews and Meta-Analyses (PRISMA) flow diagram.

The study selection process is presented in the PRISMA flow chart shown in **Figure 1**. A total of 119 studies met our inclusion criteria. The baseline characteristics of the included studies are presented in **Table 1**. A total of 130,106 patients were included, of whom 100,295 had undergone TURP, 1,530 had undergone PKRP, 90 had undergone TUIP, 4,621 had undergone OP, 3,956 had undergone HoLEP, 1,584 had undergone ThuLEP, 14,058 had undergone PVP, 217 had undergone surgery with AquaBeam, 1,796 had undergone PAE, and 1,959 had undergone TUMT procedures. Forty-two studies were RCTs, 29 were non-randomized prospective studies, and 48 were retrospective single-arm case series. Forty-nine studies were conducted in Europe, 34 were conducted in Asia, 22 studies were conducted in North America, six studies were conducted in Africa, and two studies were conducted in Oceania. In addition, another six multi-institutional studies were conducted in Europe and North America.

**Table 1:** Study characteristics.

Study	Patients (*n*)	Therapy	Study design	Setting	Country	FU (mo)
Stephenson 1991 (13)	318	TURP	RS	Database	United States	72
Sidney 1992 (14)	7,771	TURP	RS	Database	United States	96
Matani 1996 (15)	166	TURP	RS	Single center	Germany	60
Jahnson 1998 (16)	42	TURP	RCT	Single center	Sweden	60
Carter 1999 (17)	96	TURP	RCT	Single center	United Kingdom	12
Hammadeh 2000 (18)	52	TURP	RCT	Single center	United Kingdom	36
Schatzl 2000 (19)	28	TURP	NRPS	Single center	Austria	24
Keoghane 2000 (20)	76	TURP	RCT	Single center	United Kingdom	24
Floratos 2001 (21)	73	TURP	RCT	Single center	The Netherlands	36
Tuhkanen 2001 (22)	25	TURP	RCT	Single center	Finland	24
Helke 2001 (23)	93	TURP	NRPS	Single center	Germany	12
Hammadeh 2003 (24)	52	TURP	RCT	Single center	United Kingdom	60
van Melick 2003 (25)	50	TURP	RCT	Single center	The Netherlands	12
Tan 2003 (26)	30	TURP	RCT	Single center	New Zealand	12
Hill 2004 (27)	56	TURP	RCT	Multicenter	United States	60
Madersbacher 2005 (5)	20,671	TURP	RS	Single center	Austria	96
Liu 2005 (28)	32	TURP	RCT	Single center	Taiwan	24
Wilson 2006 (29)	30	TURP	RCT	Single center	New Zealand	24
Ahyai 2007 (30)	100	TURP	RCT	Single center	Germany	36
Tasci 2008 (31)	41	TURP	NRPS	Single center	Türkiye	24
Zhao 2010 (32)	102	TURP	RCT	Single center	China	36
Ou 2010 (33)	35	TURP	RCT	Single center	China	12
Muslumanoglu 2011 (34)	47	TURP	RCT	Single center	Türkiye	12
Xue 2013 (35)	100	TURP	RCT	Single center	China	36
Cui 2013 (36)	49	TURP	RCT	Single center	China	48
Mamoulakis 2013 (37)	149	TURP	RCT	Multicenter	The Netherlands, Germany, Greece, and Italy	36
Stucki 2014 (38)	67	TURP	RCT	Single center	Switzerland	12
Bachmann 2014 (39)	127	TURP	RCT	Multicenter	Austria, Belgium, France, Germany, Italy, the Netherlands, Spain, Switzerland, and the United Kingdom	6
Guo 2015 (40)	68	TURP	NRPS	Multicenter	Switzerland	60
Thomas 2015 (41)	121	TURP	RCT	Multicenter	Austria, Belgium, France, Germany, Italy, the Netherlands, Spain, Switzerland, and the United Kingdom	24
Al-Rawashdah, 2017 (42)	251	TURP	RCT	Single center	Italy	36
Eredics, 2018 (6)	20,388	TURP	RS	Database	Austria	96
Mordasini, 2018 (43)	126	TURP	RCT	Single center	Switzerland	60
Ray 2018 (44)	89	TURP	RS	Database	United Kingdom	12
Prudhomme 2019 (45)	34	TURP	RS	Multicenter	France	12
Sagen 2020 (46)	355	TURP	RS	Single center	Sweden	36
Stoddard 2021 (47)	36,040	TURP	NRPS	Database	United States	60
Abt 2021 (48)	51	TURP	RCT	Single center	Switzerland	24
Ofoha 2021 (49)	30	TURP	RS	Single center	Nigeria	12
Gilling 2022 (50)	65	TURP	RCT	Multicenter	United States, Australia, New Zealand, and the United Kingdom	60
Loloi 2022 (51)	304	TURP	RS	Single center	United States	60
Yang 2022 (52)	370	TURP	RS	Single center	China	36
Yang 2023 (53)	320	TURP	RS	Single center	China	36
Raizenne 2023 (54)	11,205	TURP	RS	Database	United States	24
Hu, 2016 (55)	467	PKRP	RS	Single center	China	60
Al-Rawashdah, 2017 (42)	246	PKRP	RCT	Single center	Italy	36
Cheng 2021 (56)	60	PKRP	NRPS	Single center	China	36
Zhu 2012 (57)	132	PKRP	NRPS	Single center	China	36
Li 2017 (58)	44	PKRP	NRPS	Single center	China	36
Elshal 2020 (59)	62	PKRP	RCT	Single center	Egypt	36
Mamoulakis 2013 (37)	146	PKRP	RCT	Multicenter	The Netherlands, Germany, Greece, and Italy	36
Wei 2016 (60)	204	PKRP	RS	Single center	China	24
Peng 2016 (61)	59	PKRP	RCT	Single center	China	12
Yip 2011 (62)	40	PKRP	RCT	Single center	Hong Kong	12
Stucki 2014 (38)	70	PKRP	RCT	Single center	Switzerland	12
Jahnson 1998 (16)	43	TUIP	RCT	Single center	Sweden	60
Elshal 2014 (63)	47	TUIP	RS	Database	Egypt	60
Sidney 1992 (14)	448	OP	RS	Database	United States	96
Eredics 2018 (6)	1,286	OP	RS	Database	Austria	96
Kuntz 2007 (64)	60	OP	RCT	Single center	Germany	60
Madersbacher 2005 (5)	2,452	OP	RS	Single center	Austria	96
Ou 2010 (33)	34	OP	RCT	Single center	China	12
Sofimajidpour 2020 (65)	80	OP	RS	Single center	Iran	12
Ofoha 2021 (49)	29	OP	RS	Single center	Nigeria	12
Varkarakis 2004 (66)	232	OP	NRPS	Single center	Greece	12
Shah 2021 (67)	94	HoLEP	RCT	Single center	India	60
Gilling 2008 (68)	71	HoLEP	NRPS	Single center	New Zealand	60
Whiting 2022 (69)	1,016	HoLEP	NRPS	Single center	United Kingdom	60
Elshal 2012 (70)	978	HoLEP	RS	Single center	Canada	60
Droghetti 2022 (71)	567	HoLEP	RS	Single center	Italy	60
Kuntz 2007 (64)	60	HoLEP	RCT	Single center	Germany	60
Enikeev 2019 (72)	127	HoLEP	RS	Single center	Russia	60
Elshal 2020 (59)	60	HoLEP	RCT	Single center	Egypt	36
Bhandarkar 2022 (73)	86	HoLEP	RCT	Single center	India	36
Vavassori 2008 (74)	330	HoLEP	NRPS	Single center	Italy	36
Ahyai 2007 (30)	100	HoLEP	RCT	Single center	Germany	36
Wilson 2006 (29)	30	HoLEP	RCT	Single center	New Zealand	24
Prudhomme 2019 (45)	17	HoLEP	RS	Multicenter	France	12
Tan 2003 (75)	30	HoLEP	RCT	Single center	New Zealand	12
Elshal 2014 (63)	50	HoLEP	RCT	Single center	Canada	12
Bae 2011 (76)	309	HoLEP	RS	Single center	Korea	12
Aho 2005 (77)	20	HoLEP	RCT	Single center	New Zealand	12
Neill 2006 (78)	20	HoLEP	RCT	Single center	New Zealand	12
Castellani 2019 (79)	412	ThuLEP	RS	Single center	Italy	12
Gross 2017 (80)	500	ThuVEP	RS	Single center	Germany	60
Tao 2019 (81)	198	ThuVEP	RCT	Single center	China	24
Tao 2017 (82)	248	ThuVEP	RS	Single center	China	24
Becker 2017 (83)	80	ThuVEP	RS	Multicenter	Italy	24
Bach 2011 (84)	90	ThuVEP	NRPS	Single center	Germany	12
Netsch 2012 (85)	56	ThuVEP	NRPS	Single center	Germany	12
Park 2017 (86)	159	PVP	RS	Single center	Korea	60
Law 2021 (87)	3,627	PVP	RS	Multicenter	Canada, France, Germany, Italy, Mexico, Brazil, and Argentina	60
Yamada 2016 (88)	1,154	PVP	RS	Single center	Japan	120
Hai 2009 (89)	321	PVP	RS	Single center	United States	60
Elshal 2014 (63)	144	PVP	RS	Database	Egypt	60
Mordasini 2018 (43)	112	PVP	RCT	Single center	Switzerland	60
Guo 2015 (40)	120	PVP	NRPS	Multicenter	Switzerland	60
Malde 2012 (90)	115	PVP	NRPS	Single center	United Kingdom	60
Ajib 2018 (91)	370	PVP	RS	Single center	Canada	60
Cheng 2021 (56)	60	PVP	RS	Single center	China	36
Kim 2016 (92)	630	PVP	RS	Single center	Korea	36
Te 2006 (93)	139	PVP	NRPS	Single center	United States	36
Tasci 2011 (94)	550	PVP	NRPS	Single center	Türkiye	36
Meskawi 2017 (95)	438	PVP	RS	Multicenter	Canada, United States, and France	36
Xue 2013 (35)	100	PVP	RCT	Single center	China	6
Guo 2015 (40)	56	PVP	NRPS	Single center	Switzerland	36
Malek 2000 (96)	55	PVP	NRPS	Single center	United States	24
Hueber 2015 (97)	1,196	PVP	RS	Multicenter	Canada, United States, France, and England	24
Tao 2013 (98)	188	PVP	NRPS	Single center	China	24
Chung 2011 (99)	162	PVP	NRPS	Single center	United States	24
Stone 2016 (100)	70	PVP	NRPS	Single center	United States	24
Liu 2020 (101)	150	PVP	RS	Single center	China	24
Campobasso 2019 (102)	1,031	PVP	RS	Multicenter	Italy	24
Tao 2019 (81)	216	PVP	RCT	Single center	China	24
Chen 2013 (103)	132	PVP	RS	Single center	Taiwan	24
Valdivieso 2016 (104)	440	PVP	RS	Multicenter	Canada, United States, United Kingdom, and France	24
Kim 2010 (105)	169	PVP	NRPS	Single center	Korea	24
Tasci 2008 (31)	40	PVP	NRPS	Single center	Türkiye	24
Ruszat 2006 (106)	183	PVP	NRPS	Single center	Switzerland	24
Ghobrial 2020 (107)	58	PVP	RCT	Single center	Egypt	24
Huet 2019 (108)	100	PVP	NRPS	Single center	France	24
Thomas 2015 (41)	128	PVP	RCT	Multicenter	Austria, Belgium, France, Germany, Italy, the Netherlands, Spain, Switzerland, and the United Kingdom	24
Prudhomme 2019 (45)	9	PVP	RS	Multicenter	France	12
Liu 2022 (109)	77	PVP	RCT	Single center	China	12
Mosli 2013 (110)	103	PVP	NRPS	Single center	Egypt	12
Seki 2008 (111)	161	PVP	NRPS	Single center	Japan	12
Hueber 2012 (112)	250	PVP	RS	Single center	Canada	12
Peng 2016 (61)	61	PVP	RCT	Single center	China	12
Tao 2019 (113)	102	PVP	RCT	Single center	China	12
Tugcu 2007 (114)	100	PVP	RS	Single center	Türkiye	12
Bachmann 2014 (39)	131	PVP	RCT	Multicenter	Austria, Belgium, France, Germany, Italy, the Netherlands, Spain, Switzerland, and the United Kingdom	6
Carter 1999 (17)	95	PVP	RCT	Single center	United Kingdom	12
Pfitzenmaier 2008 (115)	173	PVP	NRPS	Single center	Germany	12
Abolazm 2020 (116)	49	PVP	RCT	Single center	Egypt	12
Bhojani 2023 (117)	101	AquaBeam	NRPS	Multicenter	United States, Canada	60
Gilling 2022 (50)	116	AquaBeam	RCT	Multicenter	United States, Australia, New Zealand, and the United Kingdom	60
Zorn 2022 (118)	101	AquaBeam	RCT	Multicenter	United States and Canada	36
Bilhim 2022 (119)	1,072	PAE	RS	Single center	Portugal	120
Xu 2022 (120)	125	PAE	RS	Single center	China	60
Abt 2021 (48)	48	PAE	RCT	Single center	Switzerland	24
Raizenne 2023 (54)	335	PAE	RS	Database	United States	24
Ray 2018 (44)	216	PAE	RS	Database	United Kingdom	12
Gravas 2007 (121)	213	TUMT	NRPS	Single center	Greece	60
Francisca 1999 (122)	1,092	TUMT	NRPS	Multicenter	Korea, Sweden, Singapore, Spain, Canada, and the Netherlands	60
Raizenne 2022 (123)	119	TUMT	RS	Database	United States	60
Lau 1998 (124)	106	TUMT	RS	Single center	Singapore	60
Ohigashi 2007 (125)	34	TUMT	NRPS	Single center	Japan	60
Keijzers 1998 (126)	231	TUMT	NRPS	Single center	The Netherlands	60
Tsai 2000 (127)	82	TUMT	RCT	Single center	Taiwan	60
Floratos 2001 (21)	82	TUMT	RCT	Single center	The Netherlands	36

FU (mo), follow-up (months); RCT, randomized controlled trial; NRPS, non-randomized prospective study; RS, retrospective study; TURP, transurethral resection of the prostate (monopolar); PKRP, plasma kinetic resection of prostate; TUIP, transurethral incision of prostate; OP, open prostatectomy; HoLEP, holmium laser enucleation of the prostate; ThuLEP, thulium:yttrium aluminum garnet laser (Tm : YAG) enucleation of the prostate, also including ThuVEP (vapoenucleation); PVP, photoselective vaporization of the prostate; AquaBeam, image-guided robotic waterjet ablation; PAE, prostatic artery embolization; TUMT, transurethral microwave therapy.

### Risk of bias

The quality and RoB assessments are summarized in the **Supplementary Files**. For the 42 RCT studies, the RoBs of the 32 studies were considered unclear, whereas 47 of the 77 single-arm studies were assessed as having a high RoB.

### Baseline patient characteristics

As shown in **Table 2**, the preoperative characteristics of the patients were summarized and pooled in accordance with the type of procedure. It appeared that the PV, IPSS, and PVR values of patients who had undergone TUIP, OP, or TUMT were different from those of other groups. For patients who had undergone TURP, the average age was 70 years, the average PV was 55 cm^3^, the average IPSS was 22, the average PVR was 184 mL, and the average *Q*_max_ was 8 mL per s. For patients who had undergone PKRP, the average age was 69 years, the average PV was 67 cm^3^, the average IPSS was 21, the average PVR was 112 mL, and the average *Q*_max_ was 7 mL per s. For patients who had undergone TUIP, the average age was 71 years, the average PV was 26 cm^3^, the average IPSS was 16, the average PVR was 139 mL, and the average *Q*_max_ was 9 mL per s. For patients who had undergone OP, the average age was 71 years, the average PV was 106 cm^3^, the average IPSS was 24, the average PVR was 147 mL, and the average *Q*_max_ was 6 mL per s. For patients who had undergone HoLEP, the average age was 70 years, the average PV was 79 cm^3^, the average IPSS was 21, the average PVR was 186 mL, and the average *Q*_max_ was 8 mL per s. For patients who had undergone ThuLEP, the average age was 70 years, the average PV was 65 cm^3^, the average IPSS was 24, the average PVR was 138 mL, and the average *Q*_max_ was 7 mL per s. For patients who had undergone PVP, the average age was 72 years, the average PV was 63 cm^3^, the average IPSS was 22, the average PVR was 166 mL, and the average *Q*_max_ was 8 mL per s. For patients who had undergone surgery with AquaBeam, the average age was 67 years, the average PV was 79 cm^3^, the average IPSS was 23, the average PVR was 117 mL, and the average *Q*_max_ was 7 mL per s. For patients who had undergone PAE, the average age was 66 years, the average PV was 86 cm^3^, the average IPSS was 22, the average PVR was 124 mL, and the average *Q*_max_ was 10 mL per s. For patients who had undergone TUMT, the average age was 67 years, the average PV was 48 cm^3^, the average IPSS was 21, the average PVR was 76 mL, and the average *Q*_max_ was 9 mL per s.

**Table 2:** Pooled estimates for baseline confounders.

Treatment	Patients (*n*)	Age (years)	PV (cm^3^)	IPSS	PVR (mL)	*Q*_max_ (mL/s)
TURP	100,295	70	55	22	184	8
PKRP	1,530	69	67	21	112	7
TUIP	90	71	26	16	139	9
OP	4,621	71	106	24	147	6
HoLEP	3,956	70	79	21	186	8
ThuLEP	1,584	70	65	24	138	7
PVP	14,058	72	63	22	166	8
AquaBeam	217	67	79	23	117	7
PAE	1,796	66	86	22	124	10
TUMT	1,959	67	48	21	76	9

PV, prostate volume; IPSS, International Prostate Symptom Score; PVR, postvoid residual volume; Q_max_, maximum urinary flow rate; TURP, transurethral resection of the prostate (monopolar); PKRP, plasma kinetic resection of prostate; TUIP, transurethral incision of prostate; OP, open prostatectomy; HoLEP, holmium laser enucleation of the prostate; ThuLEP, thulium:yttrium aluminum garnet laser (Tm : YAG) enucleation of the prostate, also including ThuVEP (vapoenucleation); PVP, photoselective vaporization of the prostate; AquaBeam, image-guided robotic waterjet ablation; PAE, prostatic artery embolization; TUMT, transurethral microwave therapy.

### Surgical retreatments after different procedures

In **Table 3**, the surgical retreatment rates of various procedures in different follow-up years are shown. Most of the evidence was derived from studies on TURP, PKRP HoLEP, and PVP, as there were fewer studies on TUIP, OP, ThuLEP, AquaBeam, PAE, and TUMT. For almost every procedure, the risk of surgical retreatment increased over time.

**Table 3:** Surgical retreatment after different operation procedures.

	1 year		2 years		3 years		5 years	
	PI (95% CI)	Studies	PI (95% CI)	Studies	PI (95% CI)	Studies	PI (95% CI)	Studies
Resection								
TURP	4.0% (3.0% to 5.1%)	23	5.0% (3.5% to 6.6%)	14	6.0% (4.4% to 7.7%)	13	7.7% (5.8% to 9.8%)	13
PKRP	3.5% (0.6% to 8.2%)	7	3.6% (1.9% to 5.8%)	3	5.7% (3.2% to 8.8%)	6	6.6% (4.6% to 9.3%)*	1
TUIP	–	0	–	0	–	0	13.4% (6.9% to 21.5%)	2
Enucleation								
OP	1.3% (0.3% to 2.8%)	7	–	0	–	0	4.4% (1.5% to 8.7%)	4
HoLEP	2.4% (1.1% to 4.1%)	8	3.3% (0.1% to 17.2%)*	1	5.4% (3.7% to 7.2%)	5	6.6% (4.2% to 9.5%)	7
ThuLEP	3.7% (2.2% to 5.5%)	3	7.7% (4.4% to 11.8%)	3	–	0	8.4% (6.1% to 11.2%)*	1
Vaporization								
PVP	3.3% (1.8% to 5.2%)	16	4.1% (2.9% to 5.6%)	19	6.7% (4.3% to 9.5%)	9	7.1% (5.1% to 9.4%)	9
Other								
AquaBeam	2.6% (0.5% to 7.4%)*	1	3.1% (1.1% to 6.0%)	2	3.0% (0.6% to 8.4%)*	1	4.1% (1.7% to 7.2%)	2
PAE	12.2% (2.4% to 27.8%)	4	20.0% (8.9% to 34.1%)	3	26.4% (18.9% to 35.0%)*	1	23.8% (21.4% to 26.3%)	2
TUMT	9.9% (7.0% to 13.3%)	2	19.9% (15.0% to 25.7%)*	1	23.3% (16.3% to 31.2%)	3	31.2% (25.5% to 37.2%)	7

^*^Incidence rates were not pooled as only one article reported the predefined outcome.

PI, pooled incidence; CI, confidence interval; TURP, transurethral resection of the prostate (monopolar); PKRP, plasma kinetic resection of prostate; TUIP, transurethral incision of prostate; OP, open prostatectomy; HoLEP, holmium laser enucleation of the prostate; ThuLEP, thulium:yttrium aluminum garnet laser (Tm : YAG) enucleation of the prostate, also including ThuVEP (vapoenucleation); PVP, photoselective vaporization of the prostate; AquaBeam, image-guided robotic waterjet ablation; PAE, prostatic artery embolization; TUMT, transurethral microwave therapy. "-" means no data.

At 1 year, the pooled incidence of surgical retreatment was 4.0% (95% CI 3.0% to 5.1%) for the TURP cohort, 3.5% (95% CI 0.6% to 8.2%) for the PKRP cohort, 1.3% (95% CI 0.3% to 2.8%) for the OP cohort, 2.4% (95% CI 1.1% to 4.1%) for the HoLEP cohort, 3.7% (95% CI 2.2% to 5.5%) for the ThuLEP cohort, 3.3% (95% CI 1.8% to 5.2%) for the PVP cohort, 2.6% (95% CI 0.5% to 7.4%) for the AquaBeam cohort, 12.2% (95% CI 2.4% to 27.8%) for the PAE cohort, and 9.9% (95% CI 7.0% to 13.3%) for the TUMT cohort.

At 2 years, the pooled incidence of surgical retreatment was 5.0% (95% CI 3.5% to 6.6%) for the TURP cohort, 3.6% (95% CI 1.9% to 5.8%) for the PKRP cohort, 3.3% (95% CI 0.1% to 17.2%) for the HoLEP cohort, 7.7% (95% CI 4.4% to 11.8%) for the ThuLEP cohort, 4.1% (95% CI 2.9% to 5.6%) for the PVP cohort, 3.1% (95% CI 1.1% to 6.0%) for the AquaBeam cohort, 20.0% (95% CI 8.9% to 34.1%) for the PAE cohort, and 19.9% (95% CI 15.0% to 25.7%) for the TUMT cohort.

At 3 years, the pooled incidence of surgical retreatment was 6.0% (95% CI 4.4% to 7.7%) for the TURP cohort, 5.7% (95% CI 3.2% to 8.8%) for the PKRP cohort, 5.4% (95% CI 3.7% to 7.2%) for the HoLEP cohort, 6.7% (95% CI 4.3% to 9.5%) for the PVP cohort, 3.0% (95% CI 0.6% to 8.4%) for the AquaBeam cohort, 26.4% (95% CI 18.9% to 35.0%) for the PAE cohort, and 23.3% (95% CI 16.3% to 31.2%) for the TUMT cohort.

At 5 years, the pooled incidence of surgical retreatment was 7.7% (95% CI 5.8% to 9.8%) for the TURP cohort, 6.6% (95% CI 4.6% to 9.3%) for the PKRP cohort, 13.4% (95% CI 6.9% to 21.5%) for the TUIP cohort, 4.4% (95% CI 1.5% to 8.7%) for the OP cohort, 6.6% (95% CI 4.2% to 9.5%) for the HoLEP cohort, 8.4% (95% CI 6.1% to 11.2%) for the ThuLEP cohort, 7.1% (95% CI 5.1% to 9.4%) for the PVP cohort, 4.1% (95% CI 1.7% to 7.2%) for the AquaBeam cohort, 23.8% (95% CI 21.4% to 26.3%) for the PAE cohort, and 31.2% (95% CI 25.5% to 37.2%) for the TUMT cohort.

## Discussion

This systematic review comprehensively summarized the reoperation rates after surgeries for male LUTS management. We found that the retreatment rates increased over time and differed among procedures. Our results can be used to counsel both the urologists and patients regarding the different therapeutic strategies.

As the gold standard of surgical treatment for BPH/LUTSs, it was reported after a nationwide analysis of 20,671 patients that the surgical retreatment rate of TURP was 3.7% for 1 year and 9.5% for 5 years (5), which was similar to our current result. As the most widely investigated alternative to TURP and PKRP (bipolar TURP) was found to have a comparable efficacy in regard to the long-term follow-up, but was safer during the perioperative period (3). Numerous studies have reported that PKRP exhibited similar rates of surgical retreatment as TURP (3), which was consistent with our results.

Moreover, TUIP was recommended for patients with a PV of < 30 mL and those without a middle lobe (2, 3). A meta-analysis of six trials published 13 years ago showed that reoperation was more common after TUIP (18.4%) than it was after TURP (7.2%) (128). The follow-up periods of the six trials included above were different, which may introduce some bias; however, our updated review showed a similar result in that the reoperation rate of TUIP was 13.4% in 5 years, which was higher among these surgical procedures. The higher risk of surgical retreatment associated with TUIP may be due to its method, which involves only incising the bladder outlet without removing prostatic tissue. However, TUIP has been underutilized in the urological community over the years, the reasons for this include concerns related to the limitations of PV as an indicator of the need for surgery and also its long-term efficacy (129). In contrast to TUIP, during OP, the whole prostate is removed, which is recommended for patients with a PV of > 80 mL (2, 3). A nationwide analysis reported that the surgical retreatment rate of OP was 3.0% for 1 year and 6.0% for 5 years (130), which was similar to our result. Although its long-term reoperation rate seems lower than those of the other procedures, OP showed poorer perioperative safety than the other transurethral approaches, and was associated with higher rates of blood transfusions and even death (131, 132). Therefore, OP was less popular than the other minimally invasive surgeries. However, in recent years, prostatectomy with laparoscopy or robot-assisted surgery showed better safety and were also recommended by guidelines (2).

As an alternative to open enucleation, some studies reported that HoLEP has a lower risk of reoperation than TURP or PKRP (59, 133, 134), whereas another reported that there was no difference (135, 136). Indeed, our results suggest that the reoperation rates for HoLEP are similar to (and possibly slightly lower than) those for TURP or PKRP. Enucleation using another laser, ThuLEP has a rate of reoperation that is similar to (and possibly slightly higher than) that of HoLEP, which may be due to them being similar procedures. A recent interesting study from Italy reported that an improved ThuLEP technique successfully preserved the ejaculation function in most patients (137), which suggested its potential in decreasing the reoperation rates.

For vaporization, PVP has been used in clinical settings for many years and there are many related studies that have shown it has a similar efficacy to TURP (2, 3). A previous meta-analysis published by Zhou and colleagues reported that the reoperation rate after PVP was higher than that after TURP (138). However, there were only three related trials included in Zhou’s study, and the follow-up durations of these trials were different (138). Our current summarized results, which included 53 trials, reported that the reoperation rates are similar between PVP and TURP. The difference between the results of these two meta-analyses may be due to the number of articles included.

AquaBeam has come under investigation in recent years and two related trails, WATER and WATER II, reported the reoperation rate associated with it (50, 117, 118). However, there are few studies on this technique and a lack of long-term follow-up data. Although the rate of surgical retreatment appeared to be better than other procedures in our current review, whether or not AquaBeam could be an alternative to traditional procedures still needs a lot of studies and long-term follow-up to be carried out. Previous studies indicate that PAE, another surgical procedure that remains under investigation, has a higher risk of surgical retreatment than that shown in our results (9). Due to the variability of blood supply to the human prostate, non-target embolization may occur, and secondary surgical retreatment is required (139). In addition, it takes time for the prostate to shrink after vessel embolization, and PV will also stop decreasing and begin to increase after a period of time (140). Therefore, both complications and insufficient treatment response may result in a higher risk of reoperation. Overall, the efficacy and reliability of PAE remain undetermined, and further investigations and improvements are still needed. TUMT, one of the earliest technologies used for the treatment of BPH/LUTSs, has been used and studied less in recent years, due to its higher risk of retreatment and the emergence of newer, minimally invasive technologies (2, 8). Our current results confirmed that it has a higher rate of surgical retreatment. In fact, TUMT was not recommended by the latest version of the EAU guidelines, whereas the AUA guidelines still suggest that this is a reasonable approach. However, considering its higher reoperation rate and the newer, minimally invasive technologies, TUMT will likely be displaced within the next several years (2, 141).

There are some limitations or shortcomings in our current analysis and review which must be acknowledged. First, RoB was in some of the studies included through assessment. Second, our review focused only on the reoperation rates at follow-up periods of 1, 2, 3, and 5 years. However, the follow-up duration was different among studies; examples of follow-up periods were 6 months, 4 years, long term (> 5 years), and some did not last for a ‘regular’ (i.e., a multiple of a year, half a year, or 1 year) length of time. Therefore, our results are limited by the lack of data obtained during these follow-up durations. Third, 10 surgical procedures were included in our current review, of which the indication that recommended by guidelines are different. The baseline characteristics and therapeutic outcomes of patients may also have varied. Meanwhile, the great difference between data retrieved across techniques may also have led to bias. Fourth, the risk of misestimating the reoperation rate must be noted since patients lost to follow-up are common in studies. Finally, the reoperation rates of other surgical approaches excluded in our review while recommended by guidelines were also obtained during the literature search. However, as their surgical methods were outdated, less commonly used, or they were associated with a smaller number of studies, we excluded them from our current review.

In future, studies of higher quality and longer follow-up durations should be included. With the development of surgical approaches and techniques, the reoperation rate data should also be updated every few years. Meanwhile, the reoperation rate should be further refined based on its cause, and studies exploring the reason for reoperation are needed. In addition to the reoperation rate, the cost of surgical management across procedures varies, and sometimes there are even huge differences, which also affects what procedures are available for patients to choose from (142, 143). For example, a recent study reported that robotic-assisted simple prostatectomy (RASP) showed comparable efficacy and safety with a shorter hospitalization than laparoscopic simple prostatectomy (LSP) (144). However, considering the cost and unavailability of robot-assisted surgery, LSP is also a better alternative (144). Therefore, studies that evaluate the cost-effectiveness of these surgical approaches are also needed. Overall, these further investigations may lead to a reduction in the reoperation rate or prevent some common reoperation cases, which may give more information for clinical practitioners, better improve patient quality of life, and reduce medical expenses for patients.

## Conclusions

Our results summarized the reoperation rates of 10 surgical procedures over follow-up durations of 1, 2, 3, and 5 years. There was a great difference in the reoperation rate among these procedures. The OP, AquaBeam, PKRP, and HoLEP procedures exhibited a lower reoperation rate, whereas the PAE and TUMT procedures exhibited a higher rate. These data could provide reference for urologists and BPH/LUTS patients.

## Data availability statement

The original contributions presented in the study are included in the article/**Supplementary Material**. Further inquiries can be directed to the corresponding author.

## Author contributions

WH: Conceptualization, Formal Analysis, Methodology, Writing – original draft, Writing – review and editing. TD: Conceptualization, Formal Analysis, Methodology, Writing – original draft, Writing – review and editing. ZN: Software, Writing – review and editing. CH: Validation, Writing – review and editing. CL: Validation, Writing – review and editing. ZX: Data curation, Writing – review and editing. YJ: Data curation, Writing – review and editing. WQ: Funding acquisition, Supervision, Writing – review and editing.
