# Supplementary material for: Reoperation after surgical treatment for benign prostatic hyperplasia: a systematic review
Source: Front Endocrinol (Lausanne). 2023 Nov 9;14:1287212. doi: 10.3389/fendo.2023.1287212 (PMC10665564; doi:10.3389/fendo.2023.1287212)
Supplement: Supplementary file 1 [file DataSheet_1.docx]

**Supplementary material**

**Literature Search Strategy**

**Pubmed**

#1: ((benign prostatic hyperplasia[MeSH Terms]) OR (benign prostatic hypertrophy[MeSH Terms]) OR (bph) OR (Lower urinary tract symptoms) OR (benign prostatic) OR (prostatic adenoma))

#2: ((retreatment[Title/Abstract]) OR (reoperation[Title/Abstract]) OR (reintervention[Title/Abstract]) OR (secondary surgery[Title/Abstract]) OR (secondary treatment[Title/Abstract]) OR (secondary intervention[Title/Abstract]))

#1 AND #2

**Embase**

#1: (‘Hyperplasia, Prostatic’:ab,ti OR ‘Adenoma, Prostatic’:ab,ti OR ‘Adenomas, Prostatic’:ab,ti OR ‘Prostatic Adenomas’:ab,ti OR ‘Prostatic Adenoma’:ab,ti OR ‘Benign Prostatic Hyperplasia’:ab,ti OR ‘Benign Prostatic Hyperplasias’:ab,ti OR ‘Hyperplasia, Benign Prostatic’:ab,ti OR ‘Hyperplasias, Benign Prostatic’:ab,ti OR ‘Prostatic Hyperplasias, Benign’:ab,ti OR ‘Prostatic Hypertrophy, Benign’:ab,ti OR ‘Benign Prostatic Hypertrophy’:ab,ti OR ‘Hypertrophy, Benign Prostatic’:ab,ti OR ‘Prostatic Hyperplasia, Benign’:ab,ti OR ‘Prostatic Hypertrophy’:ab,ti OR ‘Hypertrophies, Prostatic’:ab,ti)

#2: (‘retreatment’:ab,ti OR ‘reoperation’:ab,ti OR ‘reintervention’:ab,ti OR ‘secondary surgery’:ab,ti OR ‘secondary treatment’:ab,ti OR ‘secondary intervention’:ab,ti)

#1 AND #2

**Web of Science**

#1: (Hyperplasia, Prostatic OR Adenoma, Prostatic OR Adenomas, Prostatic OR Prostatic Adenomas OR Prostatic Adenoma OR Benign Prostatic Hyperplasia OR Benign Prostatic Hyperplasias OR Hyperplasia, Benign Prostatic OR Hyperplasias, Benign Prostatic OR Prostatic Hyperplasias, Benign OR Prostatic Hypertrophy, Benign OR Benign Prostatic Hypertrophy OR Hypertrophy, Benign Prostatic OR Prostatic Hyperplasia, Benign OR Prostatic Hypertrophy OR Hypertrophies, Prostatic)

#2: (retreatment OR reoperation OR reintervention OR secondary surgery OR secondary treatment OR secondary intervention)

#1 AND #2

**Supplementary Figures - Risk of Bias Assessment of Included Randomized Controlled Trials**


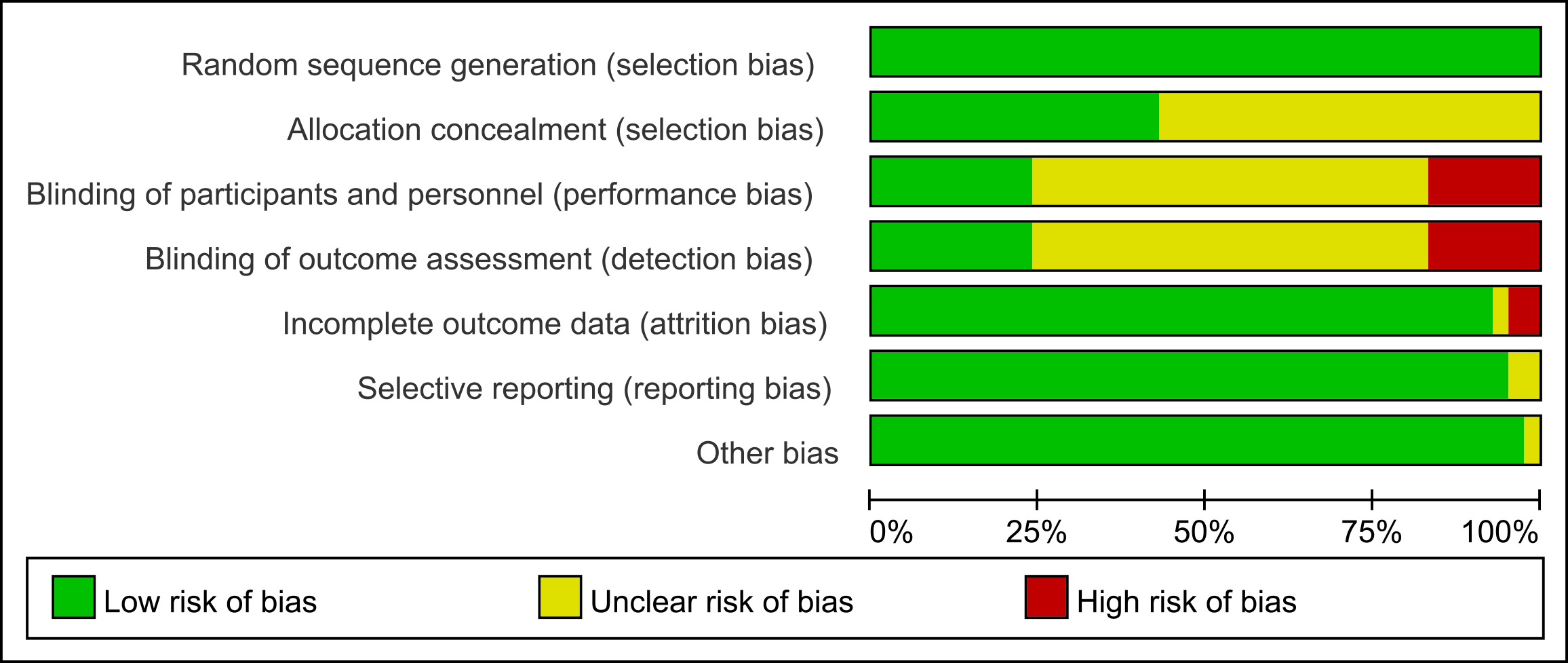

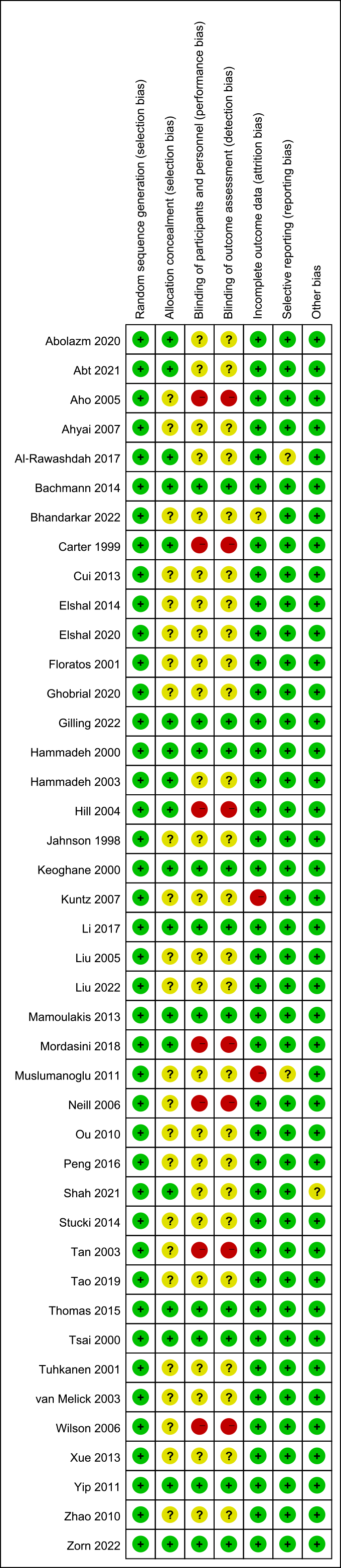


**Supplementary Table 1 - Risk of Bias Assessment of Included Single-arm Studies**

| Study | Was there an a priori protocol? | Was the total population included or were study participants selected consecutively? | Was outcome data complete for all participants and any missing data adequately explained/unlikely to be related to the outcome? | Were all prespecified outcomes of interest and expected outcomes reported? | Were primary benefit and harm outcomes appropriately measured? |
| --- | --- | --- | --- | --- | --- |
| Stephenson 1991 | Yes | Yes | Yes | Yes | Yes |
| Sidney 1992 | No | No | Yes | Yes | Yes |
| Matani 1996 | No | No | Yes | Yes | Yes |
| Keijzers 1998 | Yes | Yes | Yes | Yes | Yes |
| Lau 1998 | No | No | Yes | Yes | Yes |
| Francisca 1999 | Yes | Yes | Yes | Yes | Yes |
| Malek 2000 | No | Yes | Yes | Yes | Yes |
| Schatzl 2000 | Yes | No | Yes | Yes | Yes |
| Helke 2001 | Yes | No | Yes | Yes | Yes |
| Varkarakis 2004 | No | Yes | Yes | Yes | Yes |
| Madersbacher 2005 | No | No | Yes | Yes | Yes |
| Ruszat 2006 | No | No | Yes | Yes | Yes |
| Te 2006 | Yes | Yes | Yes | Yes | Yes |
| Gravas 2007 | Yes | No | No | Yes | Yes |
| Ohigashi 2007 | Yes | No | Yes | Yes | Yes |
| Tugcu 2007 | Yes | Yes | Yes | Yes | Yes |
| Gilling 2008 | Yes | Yes | Yes | Yes | Yes |
| Pfitzenmaier 2008 | Yes | Yes | Yes | Yes | Yes |
| Seki 2008 | Yes | Yes | Yes | Yes | Yes |
| Tasci 2008 | Yes | Yes | Yes | Yes | Yes |
| Vavassori 2008 | No | Yes | Yes | Yes | Yes |
| Hai 2009 | No | Yes | Yes | Yes | Yes |
| Jackson 2013 | Yes | Yes | Yes | Yes | Yes |
| Kim 2010 | No | Yes | Yes | Yes | Yes |
| Bach 2011 | No | Yes | Yes | Yes | Yes |
| Bae 2011 | No | Yes | No | Yes | Yes |
| Chung 2011 | No | Yes | Yes | Yes | Yes |
| Taşçı 2011 | Yes | Yes | Yes | Yes | Yes |
| Elshal 2012 | No | Yes | Yes | Yes | Yes |
| Hueber 2012 | No | Yes | Yes | Yes | Yes |
| Malde 2012 | Yes | Yes | Yes | Yes | Yes |
| Netsch 2012 | Yes | Yes | Yes | Yes | Yes |
| Zhu 2012 | Yes | Yes | Yes | Yes | Yes |
| Chen 2013 | No | No | Yes | Yes | Yes |
| Mosli 2013 | Yes | Yes | Yes | Yes | Yes |
| Tao 2013 | Yes | Yes | Yes | Yes | Yes |
| Elshal 2014 | No | No | No | Yes | Yes |
| Guo 2015 | Yes | Yes | Yes | Yes | Yes |
| Guo 2015 | Yes | Yes | Yes | Yes | Yes |
| Hueber 2015 | No | No | Yes | Yes | Yes |
| Hu 2016 | Yes | Yes | Yes | Yes | Yes |
| Kim 2016 | Yes | Yes | Yes | Yes | Yes |
| Stone 2016 | Yes | Yes | Yes | Yes | Yes |
| Valdivieso 2016 | Yes | Yes | Yes | Yes | Yes |
| Wei 2016 | Yes | Yes | Yes | Yes | Yes |
| Yamada 2016 | No | No | No | Yes | Yes |
| Gross 2017 | No | No | Yes | Yes | Yes |
| Meskawi 2017 | No | No | Yes | Yes | Yes |
| Park 2017 | Yes | Yes | Yes | Yes | Yes |
| Tao 2017 | Yes | Yes | Yes | Yes | Yes |
| Ajib 2018 | No | No | No | Yes | Yes |
| Becker 2017 | No | No | Yes | Yes | Yes |
| Eredics 2018 | No | No | Yes | Yes | Yes |
| Ray 2018 | No | No | Yes | Yes | Yes |
| Castellani 2019 | No | No | Yes | Yes | Yes |
| Huet 2019 | Yes | Yes | Yes | Yes | Yes |
| Prudhomme 2019 | No | No | Yes | Yes | Yes |
| Tao 2019 | No | No | Yes | Yes | Yes |
| Campobasso 2019 | No | No | Yes | Yes | Yes |
| Enikeev 2019 | No | Yes | Yes | Yes | Yes |
| Liu 2020 | No | Yes | Yes | Yes | Yes |
| Sagen 2020 | No | Yes | Yes | Yes | Yes |
| Sofimajidpour 2020 | Yes | Yes | Yes | Yes | Yes |
| Cheng 2021 | Yes | No | Yes | Yes | Yes |
| Law 2021 | Yes | Yes | Yes | Yes | Yes |
| Ofoha 2021 | No | No | Yes | Yes | Yes |
| Stoddard 2021 | Yes | No | Yes | Yes | Yes |
| Bilhim 2022 | No | Yes | Yes | Yes | Yes |
| Droghetti 2022 | No | Yes | Yes | Yes | Yes |
| Loloi 2022 | No | Yes | Yes | Yes | Yes |
| Raizenne 2022 | No | No | Yes | Yes | Yes |
| Whiting 2022 | No | Yes | Yes | Yes | Yes |
| Xu 2022 | No | Yes | Yes | Yes | Yes |
| Yang 2022 | No | No | Yes | Yes | Yes |
| Raizenne 2023 | No | No | Yes | Yes | Yes |
| Yang 2023 | No | No | Yes | Yes | Yes |
| Bhojani 2023 | Yes | Yes | Yes | Yes | Yes |

**Supplementary Table 2 - Baseline Patient Characteristics**

| Study | N | Therapy | Age (years) | Prostate volume (cm^3^) | IPSS | PVR (ml) | Q_max_ (ml/s) |
| --- | --- | --- | --- | --- | --- | --- | --- |
| Stephenson 1991 | 318 | TURP | 70.5 | NR | NR | NR | NR |
| Sidney 1992 | 7771 | TURP | NR | NR | NR | NR | NR |
| Matani 1996 | 166 | TURP | NR | NR | NR | NR | NR |
| Jahnson 1998 | 42 | TURP | 70.8 | 25.4 | NR | 109 | 9.5 |
| Carter 1999 | 96 | TURP | 67 | 41.7 | 21 | 135 | 9 |
| Hammadeh 2000 | 52 | TURP | 70.2 | 27 | 27 | 100 | 9 |
| Keoghane 2000 | 76 | TURP | 76 | NR | 19.4 | NR | 11.4 |
| Schatzl 2000 | 28 | TURP | NR | NR | 19.5 | 104 | 8.2 |
| Floratos 2001 | 73 | TURP | 66 | 48 | 20 | 61 | 7.6 |
| Helke 2001 | 93 | TURP | 68.7 | 49.9 | 18.3 | 102 | 8.5 |
| Tuhkanen 2001 | 25 | TURP | 67 | 55 | NR | 138 | 7.2 |
| Hammadeh 2003 | 52 | TURP | 70.2 | 27 | 26.6 | 101 | 8.6 |
| van Melick 2003 | 50 | TURP | NR | NR | 16.6 | NR | 13 |
| Tan 2003 | 30 | TURP | 70.3 | 70 | NR | NR | 8.3 |
| Hill 2004 | 56 | TURP | 66 | NR | 24.1 | 81.9 | 8.8 |
| Madersbacher 2005 | 20671 | TURP | 70.8 | NR | NR | NR | NR |
| Liu 2005 | 32 | TURP | 64.7 | 58.4 | 25.6 | 131 | 6.9 |
| Wilson 2006 | 30 | TURP | 70.3 | 70 | NR | NR | 8.3 |
| Ahyai 2007 | 100 | TURP | 68.7 | 49.9 | 21.4 | 216 | 5.9 |
| Tasci 2008 | 41 | TURP | 70.1 | 104.2 | 22.6 | 110.7 | 6.5 |
| Ou 2010 | 35 | TURP | 70.9 | 131 | 21.7 | 92.7 | 6.2 |
| Zhao 2010 | 102 | TURP | 67.8 | 67.5 | 22.4 | 97 | 8 |
| Muslumanoglu 2011 | 47 | TURP | 69.3 | 54 | 20.4 | NR | 8.3 |
| Xue 2013 | 100 | TURP | 71 | 67.3 | 23.2 | 151.1 | 8.2 |
| Mamoulakis 2013 | 149 | TURP | 68.4 | 57.1 | 23.1 | 99.2 | 8.6 |
| Cui 2013 | 49 | TURP | 70.4 | 54.8 | 20.2 | 59.8 | 8.4 |
| Bachmann 2014 | 127 | TURP | 65.4 | 46.2 | 21.7 | 109.8 | 9.9 |
| Guo 2015 | 68 | TURP | 66.4 | 44.2 | 18.4 | 95.6 | 10 |
| Stucki 2014 | 67 | TURP | 66 | 35 | 20 | 140 | 9.5 |
| Thomas 2015 | 121 | TURP | 65.4 | 46.2 | 21.7 | 109.8 | 9.9 |
| Al‑Rawashdah 2017 | 251 | TURP | 67 | 54.1 | 23.7 | 93 | 8.8 |
| Eredics 2018 | 20388 | TURP | NR | NR | NR | NR | NR |
| Mordasini 2018 | 126 | TURP | 67.6 | 37.9 | 20.4 | 114.5 | 8.5 |
| Ray 2018 | 89 | TURP | 70 | 65.6 | 21.6 | 263.6 | 10.4 |
| Prudhomme 2019 | 34 | TURP | 69.6 | NR | 14.5 | NR | 9 |
| Sagen 2020 | 355 | TURP | 74 | 50 | 20 | 725 | 8.2 |
| Abt 2021 | 51 | TURP | 66.7 | 52.1 | 17.59 | 230.7 | 7.2 |
| Ofoha 2021 | 30 | TURP | 68.5 | 59.7 | NR | NR | NR |
| Stoddard 2021 | 36040 | TURP | 70.4 | NR | NR | NR | NR |
| Gilling 2022 | 65 | TURP | 65.8 | 51.8 | 22.2 | 112 | 4.8 |
| Loloi 2022 | 304 | TURP | 69.1 | 70 | NR | NR | NR |
| Yang 2022 | 370 | TURP | 65.85 | NR | 23.04 | 97.18 | 8.93 |
| Raizenne 2023 | 11205 | TURP | 69 | NR | NR | NR | NR |
| Yang 2023 | 320 | TURP | 67.94 | 62.23 | 24.03 | 98.85 | 7.5 |
| Zhu 2012 | 132 | PKRP | 64.6 | 79.6 | 21.4 | 86.3 | 6.6 |
| Mamoulakis 2013 | 146 | PKRP | 69.3 | 58.8 | 23.3 | 92 | 8.9 |
| Yip 2011 | 40 | PKRP | NR | 61 | 21.6 | NR | 7.9 |
| Stucki 2014 | 70 | PKRP | 67 | 34 | 21 | 180 | 8.7 |
| Hu 2016 | 467 | PKRP | 70.4 | NR | 15.79 | 126.33 | 6.94 |
| Peng 2016 | 59 | PKRP | 68.7 | 64.7 | 20.4 | 84 | 7.2 |
| Wei 2016 | 204 | PKRP | 68.4 | 80.7 | 25 | 136.5 | 5.3 |
| Al‑Rawashdah 2017 | 246 | PKRP | 67.7 | 54 | 24 | 93.1 | 8.7 |
| Li 2017 | 44 | PKRP | 69.9 | 88 | 21.3 | 141.3 | 6.9 |
| Elshal 2020 | 62 | PKRP | 66.1 | 106 | 24 | 70 | 8.5 |
| Cheng 2021 | 60 | PKRP | 71.12 | 60 | 22.92 | 80.4 | 6.7 |
| Jahnson 1998 | 43 | TUIP | 70.2 | 26.2 | NR | 139 | 8.5 |
| Elshal 2014 | 47 | TUIP | 72.4 | 25.8 | 16 | NR | 8.5 |
| Sidney 1992 | 448 | OP | NR | NR | NR | NR | NR |
| Varkarakis 2004 | 232 | OP | 72.6 | 104.5 | 25 | 119 | 7.3 |
| Madersbacher 2005 | 2452 | OP | 71.4 | NR | NR | NR | NR |
| Kuntz 2007 | 60 | OP | 71.2 | 113 | 21 | 292 | 3.6 |
| Ou 2010 | 34 | OP | 71.3 | 138.4 | 23.1 | 80.3 | 5 |
| Eredics 2018 | 1286 | OP | NR | NR | NR | NR | NR |
| Sofimajidpour 2020 | 80 | OP | NR | NR | NR | NR | NR |
| Ofoha 2021 | 29 | OP | 63.8 | 65.9 | NR | NR | NR |
| Tan 2003 | 30 | HoLEP | 71.7 | 77.8 | NR | NR | 8.4 |
| Aho 2005 | 20 | HoLEP | 65.1 | 30.3 | NR | NR | 8.3 |
| Neill 2006 | 20 | HoLEP | 68.9 | 57 | 25.8 | 125 | 7.4 |
| Wilson 2006 | 30 | HoLEP | 71.7 | 77.8 | NR | NR | 8.4 |
| Ahyai 2007 | 100 | HoLEP | 68 | 53.5 | 22.1 | 238 | 4.9 |
| Gilling 2008 | 71 | HoLEP | 69.1 | 58.5 | 25.7 | 105 | 8.1 |
| Kuntz 2007 | 60 | HoLEP | 69.2 | 114.6 | 22.1 | 280 | 3.8 |
| Vavassori 2008 | 330 | HoLEP | 66 | 62 | 24 | NR | 9 |
| Bae 2011 | 309 | HoLEP | 68.3 | 55.6 | 19.1 | 72.2 | 10.3 |
| Elshal 2012 | 978 | HoLEP | 70 | 94.3 | 18.5 | 300 | 6.2 |
| Elshal 2014 | 50 | HoLEP | 71 | NR | 22.4 | 146 | 7.5 |
| Prudhomme 2019 | 17 | HoLEP | 67.1 | NR | 14 | NR | 10.5 |
| Elshal 2020 | 60 | HoLEP | 66.2 | 107 | 25 | 44 | 7.2 |
| Enikeev 2019 | 127 | HoLEP | 66.6 | 88.6 | 22 | 73 | 7.7 |
| Shah 2021 | 94 | HoLEP | NR | NR | NR | NR | NR |
| Bhandarkar 2022 | 86 | HoLEP | 67.3 | 61.1 | 20.7 | 100.5 | 8.2 |
| Droghetti 2022 | 567 | HoLEP | 69 | 80 | NR | 100 | NR |
| Whiting 2022 | 1016 | HoLEP | 72 | NR | 22 | NR | 9.4 |
| Castellani 2019 | 412 | ThuLEP | 69.8 | 58 | 26 | NR | 8.1 |
| Bach 2011 | 90 | ThuVEP | 71.3 | 108.59 | 23.46 | 178.9 | 8 |
| Netsch 2012 | 56 | ThuVEP | 69.3 | 67 | 22.08 | 114 | 9.1 |
| Gross 2017 | 500 | ThuVEP | 69 | 50 | 21 | 130 | 6.9 |
| Tao 2017 | 248 | ThuVEP | 70.7 | 76 | 27.6 | 155.3 | 6.9 |
| Becker 2017 | 80 | ThuVEP | 69.5 | 65 | 18.5 | 130 | 6.2 |
| Tao 2019 | 198 | ThuVEP | 70.7 | 86 | 24.9 | 125.3 | 8.1 |
| Cheng 2021 | 60 | PVP | 70.37 | 59.4 | 21.98 | 72.3 | 7.24 |
| Kim 2016 | 630 | PVP | 71.2 | 52.8 | 20.4 | 127 | 7.1 |
| Te 2006 | 139 | PVP | 67.7 | 54.6 | 24 | 114.3 | 7.8 |
| Taşçı 2011 | 550 | PVP | 67.6 | 64.6 | 22.8 | 124.9 | 9 |
| Meskawi 2017 | 438 | PVP | 72 | 135 | 23 | 260 | 7 |
| Xue 2013 | 100 | PVP | 72.1 | 65.8 | 23 | 148.3 | 8 |
| Ajib 2018 | 370 | PVP | 67.8 | 78.8 | 26.2 | 345 | 5.5 |
| Guo 2015 | 56 | PVP | 72 | 69 | 21 | 172 | 11.7 |
| Carter 1999 | 95 | PVP | 67.9 | 41.6 | 21 | 110 | 10 |
| Malek 2000 | 55 | PVP | 68 | 43 | 22 | 154 | 8 |
| Ruszat 2006 | 183 | PVP | 72.5 | 57.6 | 16.9 | NR | 7.1 |
| Tugcu 2007 | 100 | PVP | 67 | 98.2 | 19.2 | 122 | 6.3 |
| Pfitzenmaier 2008 | 173 | PVP | 66.8 | 45 | 20 | 104.5 | 8 |
| Seki 2008 | 161 | PVP | 71 | 51.9 | 22 | 63 | 6.8 |
| Tasci 2008 | 40 | PVP | 71.8 | 108.4 | 22.3 | 116.5 | 6.2 |
| Hai 2009 | 321 | PVP | NR | 54.7 | 24 | 169.9 | 8.6 |
| Kim 2010 | 169 | PVP | 66.5 | 42.3 | 19 | 43 | 10 |
| Chung 2011 | 162 | PVP | 72 | 91 | 18.2 | 124 | 8.3 |
| Hueber 2012 | 250 | PVP | 65.9 | 66.6 | 25 | 250 | 8.2 |
| Malde 2012 | 115 | PVP | 77.6 | 55.8 | 22 | 321 | 8 |
| Chen 2013 | 132 | PVP | NR | NR | NR | NR | NR |
| Mosli 2013 | 103 | PVP | 67.9 | 44.6 | 25.6 | 110 | 5.8 |
| Tao 2013 | 188 | PVP | 72.7 | 66 | 25.6 | 150.3 | 8 |
| Elshal 2014 | 144 | PVP | 69.2 | 29.4 | 19 | NR | 9 |
| Bachmann 2014 | 131 | PVP | 65.9 | 48.6 | 21.2 | 110.1 | 9.5 |
| Guo 2015 | 120 | PVP | 69.7 | 52.3 | 19.4 | 119.5 | 8.3 |
| Hueber 2015 | 1196 | PVP | 70 | 61.4 | 22 | 151 | 6 |
| Peng 2016 | 61 | PVP | 69.3 | 63.7 | 21.5 | 86 | 7.7 |
| Stone 2016 | 70 | PVP | 74 | NR | NR | NR | NR |
| Thomas 2015 | 128 | PVP | 65.9 | 48.6 | 21.2 | 110.1 | 9.5 |
| Valdivieso 2016 | 440 | PVP | NR | NR | NR | NR | NR |
| Yamada 2016 | 1154 | PVP | 69 | 45 | 21 | 64 | 7.4 |
| Park 2017 | 159 | PVP | 66.4 | 49.4 | 18.9 | 80.2 | 10 |
| Mordasini 2018 | 112 | PVP | 68.4 | 36.1 | 20.3 | 91.1 | 8.9 |
| Huet 2019 | 100 | PVP | 68 | NR | NR | NR | NR |
| Prudhomme 2019 | 9 | PVP | 65.6 | NR | 16 | NR | 8.6 |
| Tao 2019 | 102 | PVP | 69.5 | 81 | 28.9 | 168.2 | 6.2 |
| Tao 2019 | 216 | PVP | 72.7 | 82 | 25.4 | 132.3 | 7.6 |
| Abolazm 2020 | 49 | PVP | NR | NR | NR | NR | NR |
| Campobasso 2019 | 1031 | PVP | 69 | 60 | NR | NR | NR |
| Ghobrial 2020 | 58 | PVP | 64.5 | NR | 23 | 24 | 9.5 |
| Liu 2020 | 150 | PVP | 84.7 | 65 | 22.5 | 168.8 | 7.5 |
| Law 2021 | 3627 | PVP | 70 | 64 | 22.8 | 220.5 | 7.5 |
| Liu 2022 | 77 | PVP | 70 | 59 | 22.7 | 47.5 | 8.5 |
| Gilling 2022 | 116 | AquaBeam | 66 | 54.1 | 22.9 | 97 | 4.8 |
| Zorn 2022 | 101 | AquaBeam | 67.5 | 107.4 | 23.2 | 141 | 8.6 |
| Bhojani 2023 | 101 | AquaBeam | 67.5 | 107.4 | 23.2 | 141 | 8.6 |
| Ray 2018 | 216 | PAE | 66 | 101.2 | 21.3 | 161.6 | 8.8 |
| Abt 2021 | 48 | PAE | 65.7 | 51.2 | 19.38 | 168.5 | 7.5 |
| Bilhim 2022 | 1072 | PAE | 64.85 | 83.19 | 22.2 | 114.43 | 9.91 |
| Xu 2022 | 125 | PAE | NR | 98.43 | 22 | NR | NR |
| Raizenne 2023 | 335 | PAE | 70 | NR | NR | NR | NR |
| Keijzers 1998 | 231 | TUMT | 64.9 | 50 | NR | 64.9 | 9 |
| Lau 1998 | 106 | TUMT | 65.2 | 38.2 | NR | 86 | 9.3 |
| Francisca 1999 | 1092 | TUMT | 67 | 45 | NR | 75 | 8.7 |
| Floratos 2001 | 82 | TUMT | 68 | 42 | 21 | 34 | 9 |
| Tsai 2000 | 82 | TUMT | NR | NR | NR | NR | NR |
| Gravas 2007 | 213 | TUMT | 68 | 67.9 | 20.3 | 101.3 | 8.5 |
| Ohigashi 2007 | 34 | TUMT | 67.8 | 34.8 | 20.9 | 73 | 8.9 |
| Raizenne 2022 | 119 | TUMT | 68.5 | NR | NR | NR | NR |

NR: Not reported; Data are expressed as mean or median**.**
